# SDN Based End-to-End Inter-Domain Routing Mechanism for Mobility Management and Its Evaluation

**DOI:** 10.3390/s18124228

**Published:** 2018-12-02

**Authors:** Misumi Hata, Mustafa Soylu, Satoru Izumi, Toru Abe, Takuo Suganuma

**Affiliations:** 1 Graduate School of Information Sciences, Tohoku University, Miyagi 980-8577, Japan; izumi@ci.cc.tohoku.ac.jp (S.I.); beto@tohoku.ac.jp (T.A.); suganuma@tohoku.ac.jp (T.S.); 2 Electronics and Communication Engineering, Istanbul Technical University, 34467 Sarıyer, Turkey; mohkargan@gmail.com; 3 Cyberscience Center, Tohoku University, Miyagi 980-8577, Japan

**Keywords:** Software Defined Networking, mobility management, end-to-end routing

## Abstract

Nowadays, due to the widespread usage of mobile devices and wireless network technologies, we can use various ICT services almost anytime, anywhere even if we are changing our location at that moment. Therefore, mobility management technology have been attracting attention. This technology is to keep communication alive even when a mobile node (MN), which is communicating with the server or some nodes, moves to another network domain. Software Defined Networking (SDN) is used for mobility management to realize effective intra-domain routing that optimizes routes when an MN moves inside an SDN domain. However, many of the approaches mainly focus on intra-domain routing and it is difficult to optimize inter-domain route. In this paper, we focus on this routing optimization problem and propose an SDN based end-to-end routing mechanism specified for mobility management. The proposed routing mechanism can optimize an end-to-end route based on various parameters such as bandwidth, number of domains, and flow operations for mobility after an MN has moved across SDN domains. We carried out some simulational experimentations to evaluate the effect of proposal. It is shown that the proposed routing mechanism can reduce communication delay and enhance network performance. Thus, the proposed routing mechanism can realize effective ICT services.

## 1. Introduction

The use of mobile devices and wireless network technologies is growing rapidly. We are able to use various ICT services almost anytime, anywhere even if we are changing our location at that moment [1,2]. IEEE802.11ai is standardized [3] to have a considerable improvement in connection speed and fast authentication at access points (AP). This will expand the use of ICT services in Internet of Things (IoT) environments. However, there is an issue when a mobile device (Mobile Node: MN), which is communicating with other nodes, moves to another network domain, its IP address changes and the communication will be disconnected.

To overcome this issue, mobility management has attracted attention. Mobility management keeps communications alive between an MN which moves across network domains and its corresponding node (CN) connected. To realize mobility management, Internet Engineering Task Force (IETF) standardized Mobile IP [4,5,6]. Mobile IP enables MNs to communicate continuously while moving across network domains and it is widely used. However, there is a problem if we want to optimize route after moving. Mobile IPv4 lacks route optimization function. This might cause a communication delay and uncomfortable use of ICT services for the users due to lengthy routes. Moreover, an MN needs a specific function to use Mobile IP. Therefore not all the MN users can use Mobile IP and maintain seamless and continuous communication. Mobile IPv6 has a route optimization function but a user’s node has to have the function in addition to Mobile IPv6 and it is optional.

On the other hand, Software Defined Networking (SDN) is emerging as a new network infrastructure. SDN allows us to manage a network programmatically which grants flexible and dynamic network management. Thus, some researchers tried to apply SDN to mobility management for effective intra-domain routing that optimize routes when an MN moves inside an SDN domain [7,8]. However, they mainly focus on intra-domain routing and it is difficult to optimize an inter-domain route.

To solve this problem, we propose an SDN-based end-to-end routing mechanism specified for mobility management. The proposed routing mechanism optimizes end-to-end routes based on various parameters for mobility after an MN has moved across SDN domains. We have designed a basic inter-domain routing mechanism and showed its effectiveness [9]. This mechanism considers only the number of domain a new route goes through. The lack of information to use for selectiong a new route generate problems in quality of services after switching the route and switching time of the routing. Then, we extended our mechanism by introducing new parameters such as the number of domain hops, bandwidth between domains, and operations of flow entries [10]. However, evaluation is not enough.

In this paper, we revise the approach and evaluate our new routing mechanism through simulation experimentations. The experimental results show that our routing mechanism can reduce the communication delay and data transfer time. Therefore, it realizes effective ICT services. The contribution of this paper is to show evaluation and effectiveness of our new routing mechanism.

In the following sections, we first summarize the problems of existing mobility management approaches in Section 2. Then, we propose the mobility management specific SDN-based end-to-end routing mechanism in Section 3. Section 4 shows its architecture for implementation. We carry out some experimentations and discuss the effectiveness of the proposal in Section 5. We conclude this paper in Section 6.

## 2. Related Works

### 2.1. SDN Based Mobility Management

We introduce some approaches that apply SDN to mobility management. M. Idri uses routing header to keep communication without using IP tunneling mechanism [11]. His work does not argue about route selection. Thus, we might end up with lengthy route and also we need a master controller to manage the network that leads to an additional cost. P. Shrivastava et al. presents FastSplit that reuses previous route to reduce signaling overhead while it retains the new route near optimal [12]. The FastSplit method uses the length of path and signaling overhead to decide the new route, however it does not take real-time information into account. Y. Wang et al. proposed a Mobile IP-based mobility management that can cope with inter-domain handovers [7]. This approach uses Mobile IP for inter-domain handovers. Thus, MNs need Mobile IPv6’s route optimization function to realize route optimization but not all MNs support Mobile IPv6 or its route optimization function.

**Figure 1:**
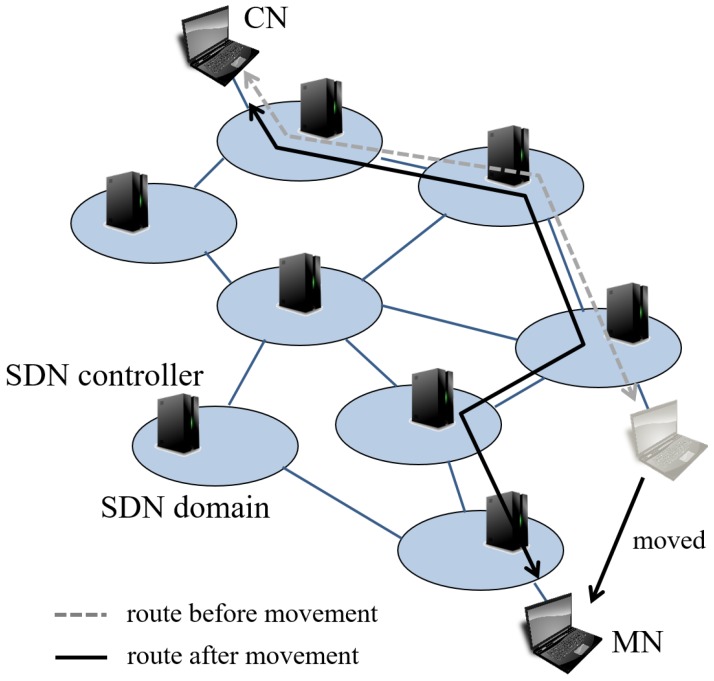
Overview of out previous work [9].

In our previous work, we proposed a SDN-based mobility management that focuses on inter-domain handovers [9]. Its overview is shown in Figure 1. Each controller manages its own SDN domain and communicate with other controllers to exchange information in need. Although we succeeded to reduce the communication delay, we had a few problems in the routing mechanism, as we did not consider route switching costs, such as the time needed to calculate a route and its flow entry installation time. To avoid disconnection, we have to update routes before a TCP session is disconnected. Therefore, we need to consider the route calculating time and the time a controller takes to add, change or delete flow entries to SDN switches, which we call flow operation, real-time network usage and processing time. Hense, we might end up with low throughput route or session disconnection. This leads to a serious low Quority of Experience (QoE) situation. Moreover, it targets a situation with only one set of communication in a network. In particular, our previous algorithm does not consider the bandwidth affected by other communications. In usual networks, many MNs communicate with their CNs. Thus, we have to consider other communications to avoid low bandwidth links.

### 2.2. Routing Approach in SDN Networks

There are several inter-domain routing mechanisms using SDN. In [13] the authors show a routing mechanism based on path cost and number of flow operations. However, this mechanism focuses on link failure due to disasters or accidents. Thus, it is not suitable for mobility management because it does not consider movement of an MN.

In [14], authors show a network model for an end-to-end QoS provisioning in inter-domain. This model selects the route mainly focusing on bandwidth. Thus, there are problems about communication delay and switching time of route for mobility management.

Phemius, K. et al. [15] proposes distributed SDN Controllers architecture. This architecture’s calculation also includes network’s information such as bandwidth. Therefore, this has problems for mobility management.

Figueira, N. et al. [16] indicates hierarchical multi-domain SDN Controllers framework. It requires a master controller to manage other SDN controllers. Thus, there is an additional cost for introducing the master controller.

## 3. End-to-End Routing Mechanism for Mobility Management

### 3.1. Overview and Design

To solve the problems we mentioned above, we propose a mobility management specific end-to-end routing mechanism. Our basic concept of routing mechanism is to consider the balance between quality of communications and the operation costs. For example, if we select a path with the fewest domain hops, it may reduce the delay but there is a risk of selecting a very low throughput route. On the other hand, if we focus on high throughput, communication delay might become larger. Requirements varies depending on what kind of service an MN user is using. If the user is using a service like video streaming, it requires a high bandwidth. If the user is using a service like chat applications that requires high performance real-time communication, it ought to require a low delay. Moreover, most importantly, we have to avoid session disconnection. Thus, the whole processing time for a handover cannot go over the total time-out limit. According to the mentioned concepts, we defined the parameters for selecting route.

- The number of domains each route candidate goes through (number of hops)
- The number of flow operations for each route candidate
- Real-time bandwidth usage information

Values of each information are normalized and weighted to be used for calculating routes.

**Figure 2:**
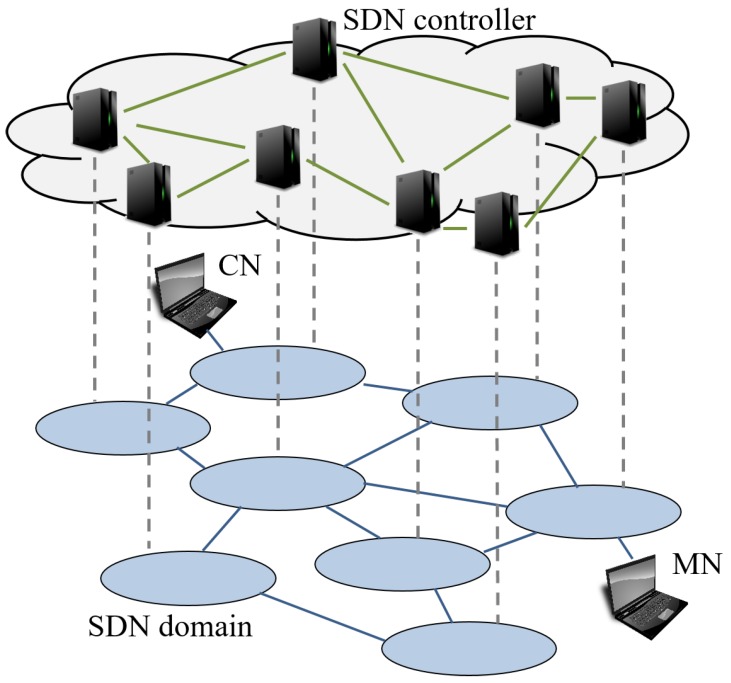
Components and networks of our mechanism.

Figure 2 indicates components and networks of our mechanism. The components include SDN controllers and SDN switches. It has two types of the networks; controller network and data network. Each SDN controller manages its own domain network and it has inter-domain information. It has the inter-domain topology information. They communicate with each other in controller network. SDN switches are connected with each other via data network.

**Figure 3:**
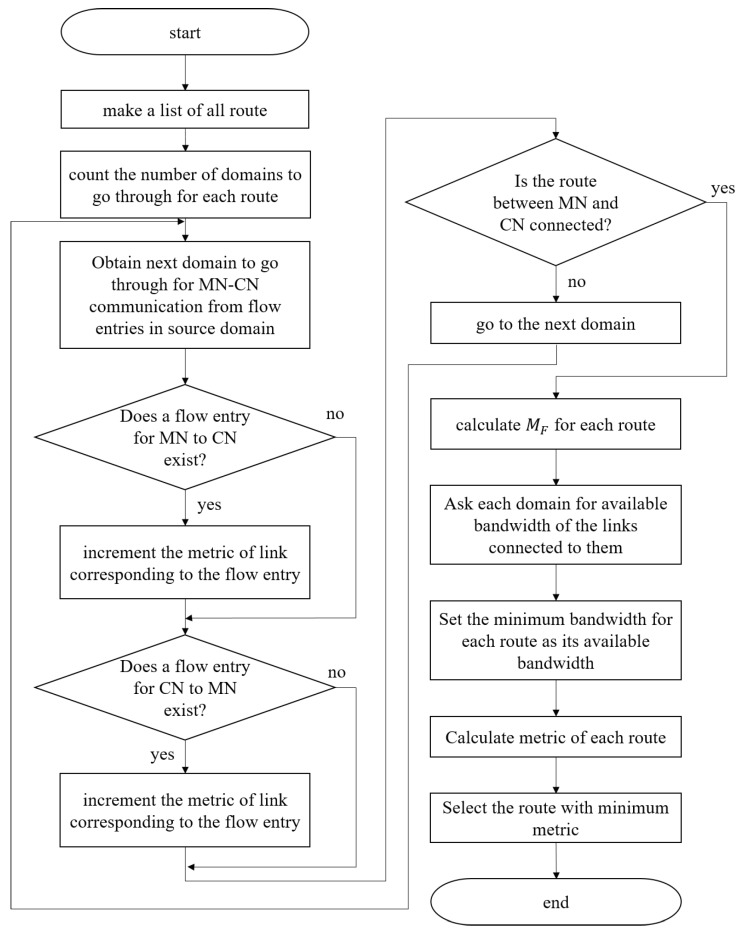
Flowchart of route calculating process.

The proposed end-to-end routing mechanism is explained in Figure 3. In this figure, source domain means the domain where an MN was, before movement. Destination domain means the domain where the MN is, after movement. The controller of the destination domain calculates the switching route. Other controllers send required information to the controller.

(1) $$M_{k}=M_{F_{k}}+M_{D_{k}}+M_{B_{k}}\;\left(k=1,2,\ldots ,K\right)$$

Equation (1) is for calculating the total value of metrics of each route we listed up. $M_{k}$ stands for the total metric of route *k*. $M_{F_{k}}$ is the total metric for flow operations, $M_{D_{k}}$ is the total metric for the number of domain hops, and $M_{B_{k}}$ is the total metric for available bandwidth of the route.

(2) $$M_{D_{k}}=\frac{D_{k}}{D_{max}}\alpha$$

(3) $$M_{F_{k}}=\frac{F_{k}}{F_{max}}\beta$$

(4) $$M_{B_{k}}=\left(1-\frac{B_{k}}{B_{max}}\right)\gamma$$

Equation (2) defines the calculation of the metric for the number of domain hops, Equation (3) defines the calculation of the metric for the flow operations, and Equation (4) defines the calculation of the metric for the available bandwidth of each route. $D_{k}$ means the number of domains route *k* goes through, $F_{k}$ means the number of flow operations for switching route *k*, and $B_{k}$ shows the minimum bandwidth of route *k*. $D_{max}$ means the number of domains in the network and $B_{max}$ means the largest bandwidth in the network. $F_{max}$ shows 2N when we set the number of domains in a network as *N*. This is because we need to set flow entries for both ways for communication between an MN and a CN. α, β, and γ are weights to take balance among each parameter.

Our routing mechanism calculates $M_{k}$ for distinct routes and select route *k* when $M_{k}$ is minimum. Based on this process, our routing mechanism can select effective route for mobility management to keep seamless communication with a low delay.

### 3.2. Example of Routing

**Figure 4:**
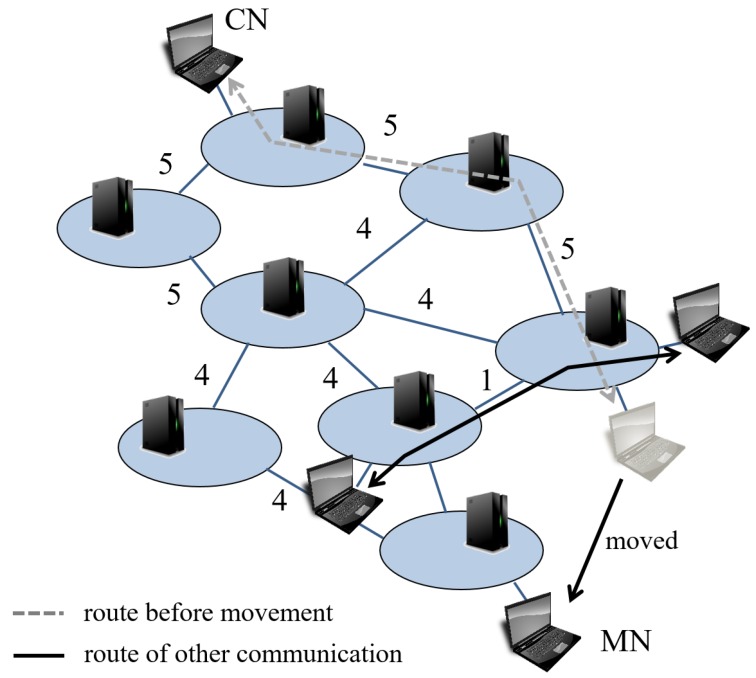
Example of network.

**Figure 5:**
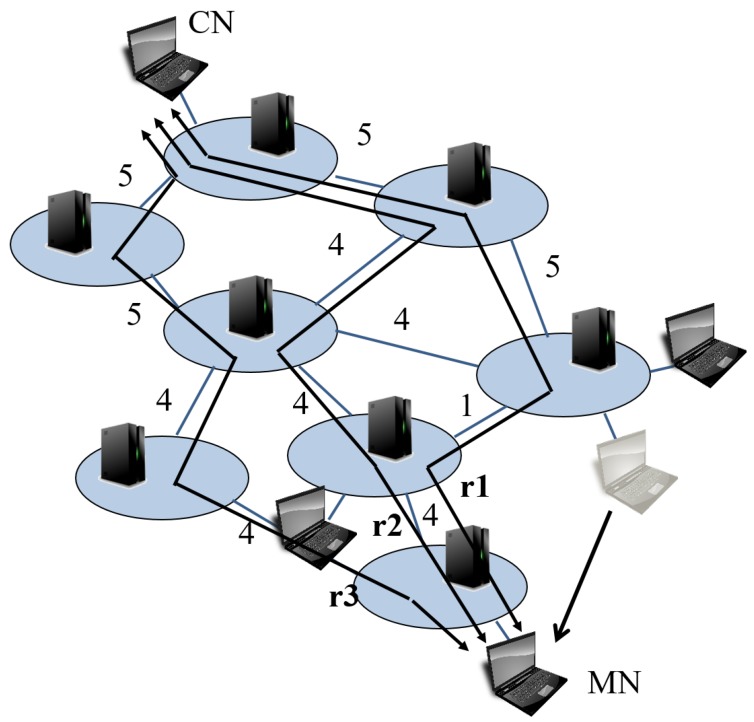
Lists of routes.

An example of the routing process is explained according to the flowchart. Figure 4 indicates an example of network. The numbers near links are their bandwidth. When an MN, which is communicating with a CN, moves to another domain, the controller of the destination domain detects the movement. Then, the controller creates a list of all possible routes from the MN to the CN. Figure 5 shows the routes’ list. There are many possible routes from the MN to the CN, but for convenience, we list up 3 routes; route 1 (r1) to route 3 (r3). The proposed routing mechanism calculates the metric $M_{k}$ for each route. In this case, $F_{max}$ is 16, $D_{max}$ is 8, and $B_{max}$ is 5. Also we set weights α, β, and γ as 1.

(5) $$M_{1}=M_{F_{1}}+M_{D_{1}}+M_{B_{1}}=\frac{6}{16}+\frac{5}{8}+\left(1-\frac{1}{5}\right)=1.80$$

(6) $$M_{2}=M_{F_{2}}+M_{D_{2}}+M_{B_{2}}=\frac{8}{16}+\frac{5}{8}+\left(1-\frac{4}{5}\right)=1.33$$

(7) $$M_{3}=M_{F_{3}}+M_{D_{3}}+M_{B_{3}}=\frac{10}{16}+\frac{5}{8}+\left(1-\frac{4}{5}\right)=1.45$$

In this example, $M_{2}$ is minimum, thus, routing mechanism selects route 2. As mentioned in the example, our routing mechanism can select effective route to take balance among number of domain to go through, flow operations, and available bandwidth.

## 4. Implementation

**Figure 6:**
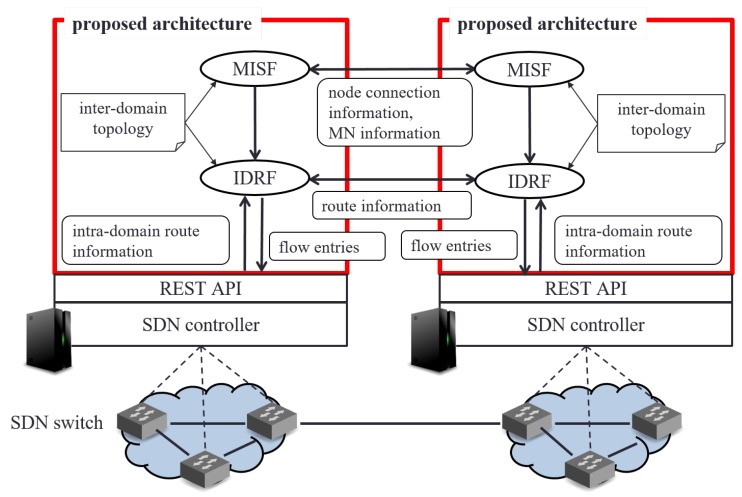
Basic architecture of our proposal.

We indicate implementation of the basic architecture with our routing mechanism based on SDN. Figure 6 is its architecture. In this architecture, we introduce two modules into SDN controllers; Management Information Sharing Function (MISF) and Inter-domain Routing Function (IDRF).

All the SDN controllers in a network have an inter-domain topology information. The inter-domain topology information consists of network address of each domain in network, IP address of each domain’s SDN controller, and inter-domain link information. MISF and IDRF use this information to search domains and calculate routes.

**Figure 7:**
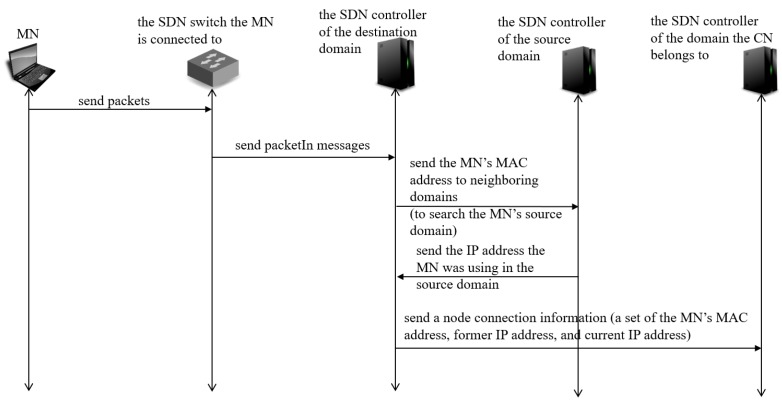
Sequence diagram of MISF.

**Figure 8:**
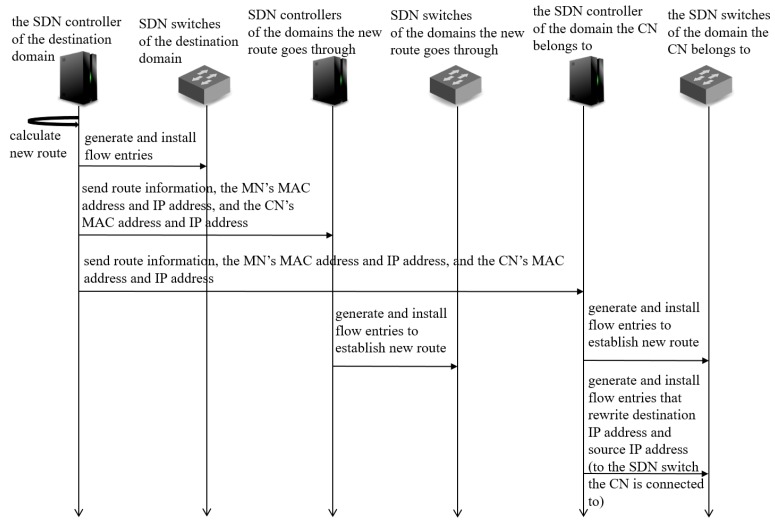
Sequence diagram of IDRF.

- Management Information Sharing Function (MISF)
  - Figure 7 shows a sequence diagram of MISF. When an MN makes an inter-domain move, the SDN controller of the destination domain detects the connection of the MN within the destination domain (attachment of the MN). The SDN controller detects the attachment of the MN with the packets sent from the MN which the SDN controller of the destination domain does not have information of the MN such as MAC address and IP address. Then, the SDN controller of the destination domain searches for the MN’s source domain by asking from the destination domain’s neighboring domains whether a node with the MN’s MAC address belonged to them. If there was, the SDN controller of the source domain replies the IP address the MN was using in the source domain. After discovering the source domain, the SDN controller of the destination domain informs a node connection information, that is a set of the MN’s MAC address, the IP address the MN was using in source domain (former IP address), the IP address the MN is using in destination domain (current IP address) to the SDN controller of the domain the CN belongs to. The SDN controller obtains the information of the domain the CN belongs to from the packets sent from the MN to the CN.
- Inter Domain Routing Function (IDRF)
  - Figure 8 shows a sequence diagram of IDRF. The SDN controller of the destination domain does all the calculation and the queuing. This function selects a new route using the routing mechanism we described above. Then, it informs the SDN controllers of the domains the new route goes through a bundle of information needed for establishing the new route; the MN’s MAC address, current IP address, former IP address, the CN’s MAC address and IP address. The SDN controllers that received them generate and install appropriate flow entries into SDN switches. In addition, in the domain which the CN belongs to, this function generates flow entries that rewrites destination IP addresses and source IP addresses of the packets sent between the MN and the CN and installs them in the SDN switch the CN is connected to. The rewriting of IP addresses is based on the node connection information that it received in the MISF. SDN switches do this rewriting processes. This allows the MN and the CN to continue their communication by keeping TCP connection.

The MISF shares information of MNs and domain topology information among controllers. The controllers exchange these kinds of information for inter-domain handover processes. The IDRF exchanges route information and calculates switching route according to the proposed routing mechanism.

For SDN controllers, we use OpenDaylight [17] and we use Open vSwitch [18] for SDN switches. We use OpenFlow Protocol ver.1.3 [19] for communication between SDN controllers and SDN switches. We get flow entry information and network information from SDN controllers via REST API as the interface of the proposed routing mechanism.

## 5. Evaluation

### 5.1. Overview

We carried out experiments to verify (1) the effectiveness of our approach compared with other approaches and (2) the effects of various weights on network throughput and the communication delay between an MN and a CN. We also measured the time it takes to calculate a new route and set flow entries (processing time) for the proposed approach and the previous approach.

To evaluate our new approach, we compared with our previous approach and a Mobile IP-like approach. Mobile IP-like approach sets a route that transfers from the domain that the MN was in before the MN’s movement to current domain. As its process is different from Mobile IP, we did not measure the processing time for this approach.

We built a virtual network with Mininet 2.2.1. The bandwidth capacities of links in the grid part are set randomly from 100 Mbps, 200 Mbps, 300 Mbps and 400 Mbps. Appearance ratio of each bandwidth is 10%, 20%, 30%, and 40% relatively. This means that 10% of all links have 100 Mbps bandwidth, 20% of them have 200 Mbps, 30% of them have 300 Mbps, and 40% of them have 400 Mbps, respectively. We also set every link except the ones connected to the MN and the CN 0.5 msec. delay. The links connected to the MN and the CN has 1000 Mbps bandwidth and 0 msec. delay. In the experiments, we aim to explore the characteristics of our routing mechanism. Thus we do not give detailed limitation range of each variable. We set range of each variable to positive integer.

The experiments are conducted in the following scenario:

1. An MN, that is communicating with a CN moves to its neighboring SDN domain.
2. Select a new route and set flow entries.
3. After changing route, send 1000 MB data from the MN to the CN and measure the transfer time.
4. Then, send 50 pings from the MN to the CN and measure the average RTT.

We executed four trials in each experiment. In each trial, the experimental network is generated automatically with random bandwidths.

### 5.2. Experiment 1: Comparison with Legacy Approaches

**Figure 9:**
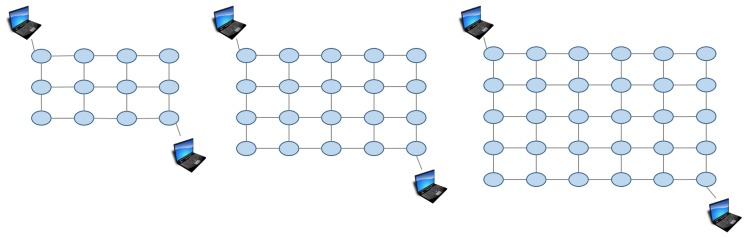
Virtual network in Experiment 1.

In the first experiment, we verified the effect of the proposed method compared to the Mobile IP-like approach and the previous approach in various sized networks. We prepared three sized grid networks; 3 by 4, 4 by 5, 5 by 6. They are shown in Figure 9. In this experiment, we set the values of α,β,γ in the proposed approach as the default ($C_{D}=1,C_{F}=1,C_{B}=1$), no weights put on specific features.

**Figure 10:**
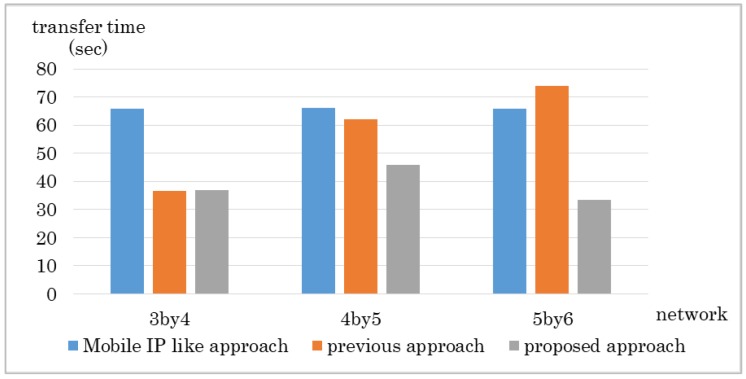
Experiment result: Data transfer time in Experiment 1.

**Figure 11:**
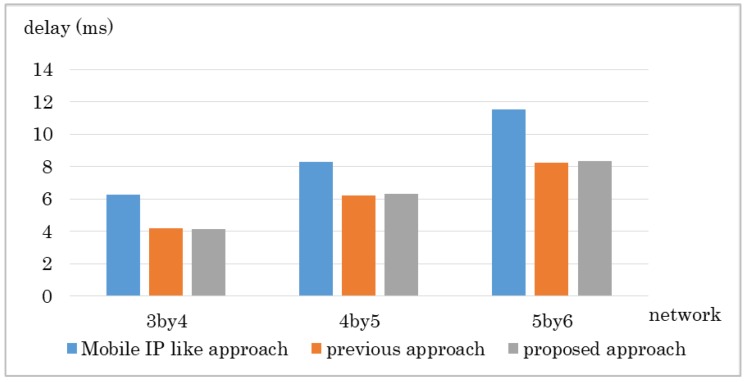
Experiment result: Communication delay in Experiment 1.

The results are shown in Figure 10 and Figure 11. The proposed method got the shortest transfer time in every network size. The delay for the proposed approach was lower than the Mobile IP-like approach but about the same as the previous approach. The Mobile IP-like approach’s route cannot be the shortest route in this case because the MN moves nearer to the CN however, the route has to go through the home domain. The previous approach considers number of hops first therefore it chooses the route with least hops, which results in the least delay. However, it does not consider bandwidth so if there is any link with small bandwidth in the route it chose, it ends up having a low throughput. The Mobile IP-like approach and the previous approach does not consider bandwidth. Therefore, even if there is a link that has small bandwidth in the route they choose, they cannot avoid the route.

### 5.3. Experiment 2: Effects of Weights

**Figure 12:**
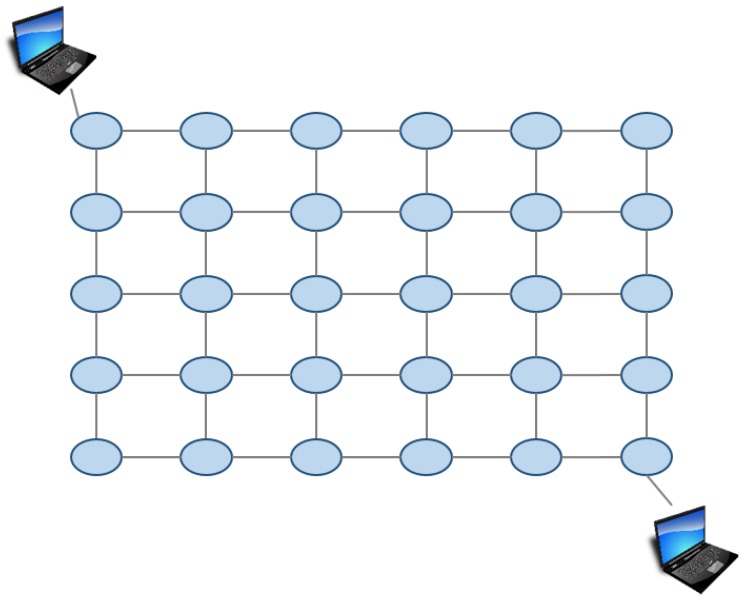
Virtual network in Experiment 2.

In the second experiment, we changed the three weights and investigated how the changes affect throughput, delay, and processing time. We used the 5by6 grid network shown in Figure 12 in this experiment. The weights α,β,γ is two-valued in this experiment. 5 for higher weight, 1 for lower weight.

**Figure 13:**
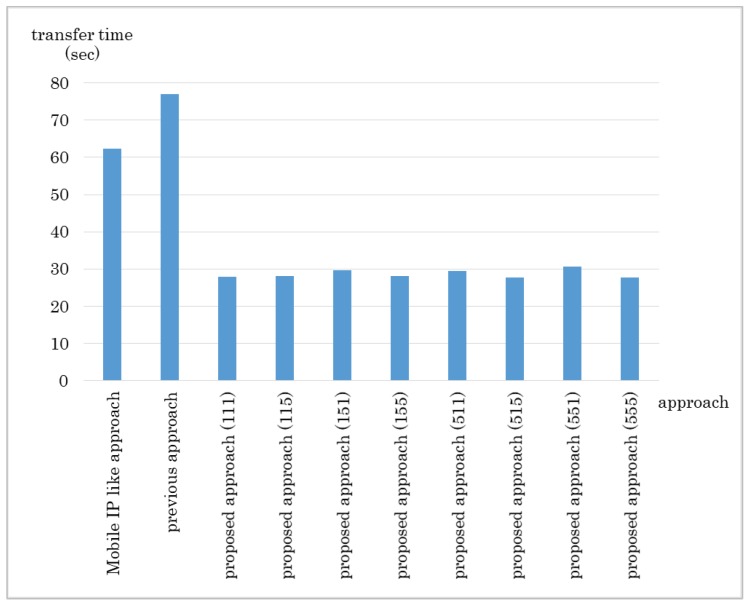
Experiment result: Data transfer time in Experiment 2.

**Figure 14:**
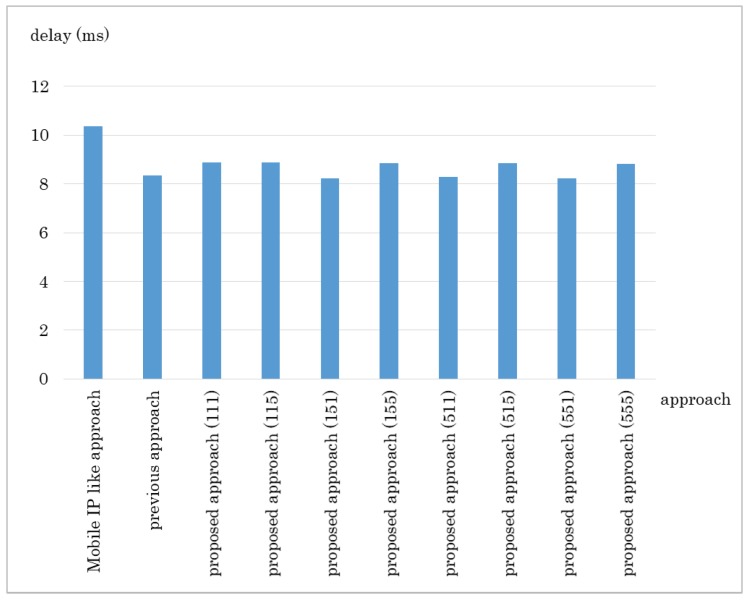
Experiment result: Communication delay in Experiment 2.

The results are shown in Figure 13 and Figure 14. As we can see from Figure 13, the proposed method improves the Mobile IP-like approach and the previous approach with any combination of weights. The delay seems to differ a bit even so, it was mostly the same when we investigated each trial independently. However, there was a particular network that the delay differed depending on the combination of metrics.

**Figure 15:**
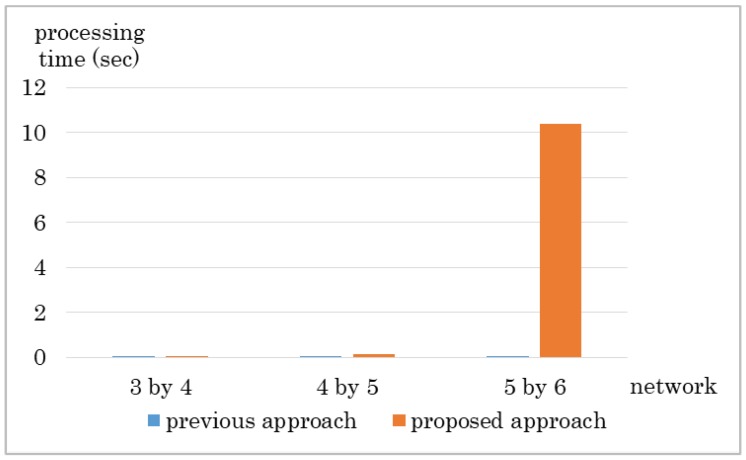
Experiment result: Processing time.

In Figure 15 we show the processing time for calculating the route. Our proposed mechanism can calculate route in shorter time. However, as network size becomes larger, processing time is longer. Thus, we need to revise the algorithm to reduce calculation time.

### 5.4. Discussion

The number of flow entries had almost no affect on the results though, there was no overloaded or low performance SDN controllers and SDN switches. However, in real environments, there are possibilities of using low performance or overloaded SDN controllers and SDN switches. In such a network, the number of flow entries should affect more to the processing time since it takes more time to calculate and install necessary flow entries with such devices.

As a reference, the default number of times TCP retries to retransmit a data before it disconnects its session is five, and the minimum Retransmission Time Out (RTO) is 0.2 s. for Linux. RTO is doubled after each retransmission hence the time limit for changing a route is approximately 6 s minimum. As this is a theoretical value and not a real value, we take this value for reference. In a scenario where the time-out limit is 6 s., the proposed approach cannot finish changing route before the time-out limit in the 5 by 6 network.

We have to note that the processing time we measured does not include the time the controller takes to detect the attachment of an MN or the time it takes for SDN controllers to install flow entries into SDN controllers in real network environment. We focused only on the processing time of the route calculation and setting flow entries in this paper. In future, we would like to evaluate in real network environment to measure the time for the whole process from the start of a movement of an MN to the end that all flow entries are installed and the MN starts communicating with a new route.

## 6. Conclusions

In this paper, we proposed a routing mechanism for mobility management and showed the detailed design. This routing mechanism can communicate with low-delay route after inter-domain handovers and avoid disconnection that might occur when switching the route. We also conducted experimentations to show effectiveness of our routing mechanism.

For future works, we have a plan to extend our routing mechanism further by implementing features such as calculation of weights and addition of weight to parameters based on the results. Furthermore, we will try to update to the lexicographic approach based on the paper [20]. Also we will carry out experiments in actual network environments.
